# Fabrication of Alginate/Ozoile Gel Microspheres by Electrospray Process

**DOI:** 10.3390/gels10010052

**Published:** 2024-01-11

**Authors:** Gianluca Ciarleglio, Tiziana Russo, Elisa Toto, Maria Gabriella Santonicola

**Affiliations:** 1 Department of Chemical Engineering Materials Environment, Sapienza University of Rome, Via del Castro Laurenziano 7, 00161 Rome, Italy; gianluca.ciarleglio@uniroma1.it (G.C.); trusso@ibecbarcelona.eu (T.R.); elisa.toto@uniroma1.it (E.T.); 2 Erbagil s.r.l., Via Luigi Settembrini 13, 82037 Telese Terme, Italy

**Keywords:** electrospray, alginate microsphere, drug delivery

## Abstract

Natural polymers, such as alginate and chitosan, are widely exploited for drug delivery applications due to their biocompatibility, low toxicity, and sustainable sourcing. In this study, pH-responsive gel microspheres were fabricated from an alginate/Ozoile emulsion. Ozoile (Stable Ozonides) is a biological inducer, derived from olive oil, which stimulates the endogenous defense system by promoting the repair of tissue damage and restoration of proper physiology through the regulation of gene transcription. Here, the versatile and cost-effective electrospray technique without the use of organic solvents was used to fabricate alginate/Ozoile microspheres with high throughput. The process parameters (voltage, flow rate, and needle gauge) were optimized to obtain microspheres with good sphericity factor and tailored diameter (250–700 μm). The microspheres were additionally optimized through a chitosan coating to enhance their stability and regulate the gel matrix’s degradation process. Morphological analysis, FTIR spectroscopy, and degradation tests confirmed the structural integrity and pH-responsive behavior of the gel microspheres. This research offers a promising route for targeted drug delivery systems, particularly in applications related to the modulation of oxidative stress and management of inflammation.

## 1. Introduction

Gels based on natural polymers are extensively used in a wide range of biomedical applications, such as tissue engineering, scaffolds, and drug delivery [1,2,3]. These gels exhibit a three-dimensional network capable of absorbing and retaining large amounts of water and have exceptional biocompatibility, biodegradability, and renewability [4,5]. Alginate, a polysaccharide extracted from brown algae, is a highly hydrophilic biopolymer commonly used in the preparation of hydrogel-based systems for drug delivery and therapeutic applications, including cancer therapy [6,7,8]. For these applications, the ability to control the gelation of alginates is crucial and depends on several factors, such as molecular composition (the ratio of mannuronic to guluronic acid), molecular weight, and the presence of a gelling agent (e.g., Ca^2+^ ions) [9]. Moreover, alginate is renowned for its biocompatibility, ready availability, biodegradability, water solubility, minimal immunogenicity, and low toxicity [10]. Following drug release, the alginate breaks down into water-soluble oligomers, which are subsequently metabolized and excreted from the body, owing to their biodegradable nature.

Alginate microspheres (MS) fabricated by electrospray techniques are used in controlled drug delivery systems. Electrospray (ES) is an electrohydrodynamic fabrication method in which a polymer solution is forced through a nozzle and, under the influence of an electric field, droplets are formed [11]. This technique provides a versatile and cost-effective means of fabricating these microspheres with high throughput, yielding products with tailored diameters and sphericity. By optimizing the process parameters, such as voltage, flow rate, and needle size, it is possible to precisely control the size and morphology of the microspheres, ensuring that they meet specific requirements for drug delivery systems. For instance, Hsu et al. used the electrospray method to fabricate doxorubicin-poly (lactic-co-glycolic acid) (PLGA) microspheres for cancer treatment [12], and Liu et al. developed a hierarchical electrospraying technique to fabricate composite PLGA and chitosan (CS) microspheres capable of loading multiple drugs for multistage release in clinical treatment [13].

pH-sensitive alginate microspheres have great potential for the delivery and controlled release of fat-soluble active ingredients such as Ozoile. Ozoile is a pool of molecules produced in a patented process by reaction of a defined oxygen–ozone mixture with the olefinic bonds of organic olive oil, which leads to the formation of stable ozonides [14]. Specifically, the process involves the 1,3 dipolar cycloaddition of ozone, obtained from pure oxygen through a 10 kV electrical discharge, which interacts with the olefinic bonds of olive oil according to the Criegee mechanism [15]. From the biological point of view, Ozoile acts as an inducer capable of modulating the main metabolic pathways. Topical treatments with Ozoile in patients with balanitis xerotica obliterans (BXO) have shown an anti-inflammatory and tissue-regenerating effect by reducing the mRNA levels of pro-inflammatory cytokines IL-1β, TNF-α, INF-γ, transglutaminase 2 and NOS2, as well as increasing the expression of hypoxia-inducible factor (HIF)-1alpha, vascular endothelial growth factor (VEGF), and E-cadherin gene expression [16]. Ozoile showed a decrease in LPS-induced inflammatory response in colonic epithelial cells and THP-1 monocytes [17]. Furthermore, in another study on patients with phlebostatic leg ulcers, Ozoile was shown to stimulate fibroblast proliferation and neoangiogenesis [18,19]. While the beneficial effects of Ozoile have been reported, in these studies no cytotoxic effects or sensitization with allergic reactions are described. To the best of our knowledge, there are no known side effects of Ozoile in the scientific literature.

In this work, pH-responsive alginate gel microspheres were fabricated by electrospray technique without the use of organic solvents. An emulsion-based approach was used to incorporate Ozoile into pH-responsive microspheres to enhance its bioavailability at targeted inflammatory sites for therapeutic purposes. The fabrication of Ozoile-containing microspheres was developed, and the electrospray process parameters were optimized to obtain microspheres of small size and spherical shape. Small-sized microspheres have a greater capacity to deliver drugs and ensure a more uniform distribution of therapeutic agents in the body, ultimately increasing treatment efficacy [20]. Chitosan-coated Ozoile-containing microspheres were also prepared to enhance their pH responsiveness.

Various techniques were employed to study the properties of the microspheres. The size and morphology of the microspheres were evaluated using optical microscopy and ImageJ software (v. 1.53). The chemical composition of the MS was examined using Fourier transform infrared spectroscopy (FTIR), and differential scanning calorimetry (DSC) was used to assess thermal stability. Degradation tests at different pH values (2, 4, 7.4, 10) at a temperature of 37 °C were conducted to evaluate the pH-responsive behavior of the Ozoile-containing microspheres. The role of chitosan coating was also analyzed to assess its influence on polymer mesh degradation.

The results in terms of morphology, thermal stability, swelling, and pH-responsive behavior are useful in establishing the potential use of Ozoile-containing MS at specific inflammatory sites.

## 2. Results and Discussion

### 2.1. Optimization of MS Fabrication by Electrospray

**Figure 1:**
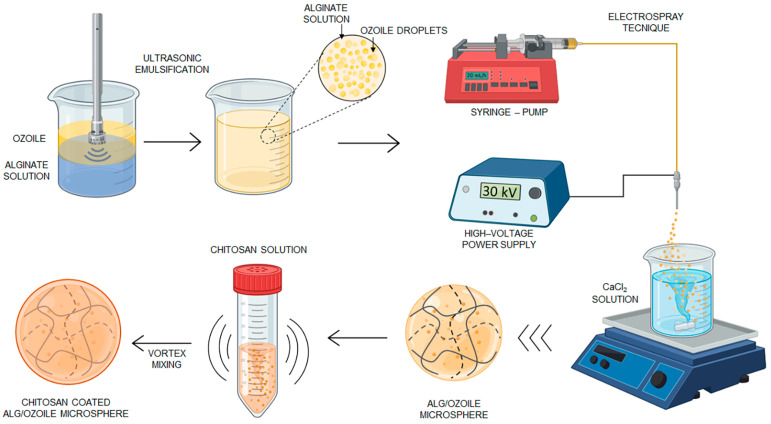
Schematic representation of the production process of pH-responsive microspheres by electrospray technique coated with chitosan involving vortex mixing.

The fabrication process of Alg/Ozoile microspheres was developed and optimized using the electrospray technique without the use of organic solvents through ionic gelation. This process is schematically represented in Figure 1.

**Figure 2:**
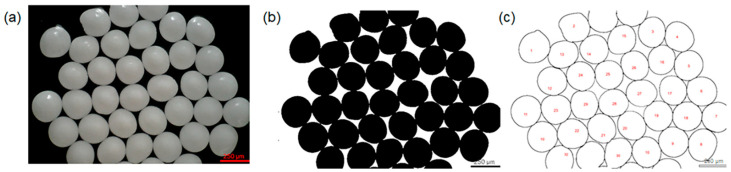
Sequence of image analysis by ImageJ software. (**a**) Optical microscopy image of Alg/Ozoile (20 wt%) microspheres prepared by electrospray (flow rate 20 mL/h, voltage 30 kV, needle size 24 G), (**b**) 8-bit conversion and binarization, (**c**) microsphere shape assignment and numbering on the binarized optical image.

The optimization of process parameters was carried out by individually varying the electrospraying parameters, including flow rate, applied voltage, and needle size. In the initial phase of experimentation, an emulsion with a 20 wt% Ozoile concentration was used. During this stage, the influence of these process parameters on the morphology and size of the microspheres was evaluated. To assess the morphology of the MS, an analysis by optical microscopy was conducted as shown in Figure 2a. In addition, a dimensional analysis was conducted on 50 samples using the ImageJ software: the images were first made binary (Figure 2b) and then the mean diameter of the microspheres was measured using the “Analyze particles” tool (Figure 2c).

The image processing was repeated for all obtained microspheres by varying the process parameters. The results, in terms of mean diameter, are shown in Table 1.

From the collected experimental data, the effect of the process parameters on the MS mean diameter was evaluated. Increasing the voltage from 25 kV to 30 kV, a reduction in the microsphere size was observed. This could occur because a higher voltage generates a higher electrostatic force between the needle and the collecting substrate, causing the droplets to break into smaller microspheres during the electrospray process. Higher voltage leads to a greater dispersion of the droplets into smaller particles. Reducing the flow rate from 30 mL/h to 20 mL/h leads to a decrease in the size of the MS. This phenomenon is associated with a reduction in the amount of emulsion delivered during electrospraying. A lower flow rate allows for more controlled droplet formation, thus contributing to the production of smaller and more uniform microspheres. Finally, using a needle with a smaller inner diameter (24 G, 0.311 mm) produces smaller microspheres than using a needle with a larger inner diameter (21 G, 0.514 mm). This is because a needle with a narrower inner diameter allows for better fragmentation of the droplets into microspheres during electrospraying. A smaller inner diameter also allows for better control over droplet formation, contributing to the production of smaller microspheres.

Three-way analysis of variance (ANOVA) was used to conduct the statistical analysis to evaluate whether the effect of process parameters on the MS size was statistically significant; Table 2 shows the results. ANOVA unveiled that needle diameter, flow rate, and applied tension show a significant impact on the microsphere size. Low flow rate and high applied tension seem to be the optimal conditions for obtaining smaller microspheres. In addition, the interactions between these factors were examined, and it was found that these interactions also play a significant role in influencing microsphere size. For example, the combination of needle diameter and applied tension or flow rate and applied tension can have synergic effects on the microsphere size.

Overall, the ANOVA results highlight the complexity of the electrospraying process and its sensitivity to control parameters. 

Figure S1 provides a graphical representation of the results of the three-way ANOVA analysis on the mean values of microsphere diameter influenced by the parameters needle size, applied voltage, and flow rate of the electrospraying process.

Table 3 presents the results of the experimental studies on the effects of different process parameters on the sphericity of alginate microspheres containing 20 wt% of Ozoile. The considered process parameters are the needle size, flow rate, and applied voltage. The sphericity of the microspheres is measured in terms of a sphericity factor (SF).

The results of the ANOVA are summarized in Table 4 and provide information on which process parameters significantly affect the sphericity of the microspheres. In this case, the flow rate and applied voltage have a significant impact on microsphere sphericity, while the needle diameter does not appear to have a significant effect. Additionally, some interactions between the parameters were examined, but only the interaction between needle diameter and applied voltage seems to significantly influence sphericity.

In summary, the analysis of the process parameters used for the fabrication of Alg/Ozoile microspheres demonstrated that the optimal process conditions for achieving both reduced microsphere size and enhanced sphericity are as follows: applied voltage of 30 kV, flow rate of 20 mL/h, and the use of a 24 G needle with internal diameter of 0.311 mm. These parameters lead to microspheres with diameter within the size range of 200–300 µm, which could provide a more sustained release and prolonged therapeutic effects, based on literature reports [21,22].

We focused not only on optimizing the microsphere fabrication process but also on the critical phase of coating these microspheres with chitosan. During the coating process, the interaction of functional groups NH_3_^+^ from chitosan and COO^−^ from alginate resulted in the formation of ionic bonds, adding a layer of functionalization to the microspheres. In particular, we investigated the influence of stirring speed on the size of alginate and Ozoile microspheres. Stirring speeds ranging from 500 to 700 rpm were employed, and the microsphere sizes were determined through image analysis utilizing ImageJ software. As shown in Table 5, the results revealed a trend: higher stirring speeds are associated with lower microsphere diameters, while water content and swelling ratio are almost constant at different rpm values.

Among the tested stirring speeds, 700 rpm was selected for the production of smaller beads. This choice is aligned with previous findings in the literature, where coating processes involving stirring have been observed to potentially induce microsphere shrinkage. In our case, this phenomenon can be attributed to the diffusion of chitosan molecules within the alginate matrix. This diffusion leads to a partial collapse of the polymer network due to electrostatic neutralization, resulting in a reduction in the size of the chitosan-coated microspheres. 

After fine tuning the process parameters, we turned our focus towards system parameters, particularly by varying the Ozoile concentration within the microspheres, ranging from 0 to 50 wt%.

### 2.2. Morphology and Swelling Properties of Alginate/Ozoile MS

Table 6 presents the mean diameters of the gel microspheres at various Ozoile concentrations from 0 to 50 wt%. The dimensional analysis revealed that there are no statistically significant differences in the size of the electrosprayed microspheres upon changing Ozoile concentration. The histograms with the diameter distribution of the Alg/Ozoile MS, with and without chitosan coating, are reported in Figures S2 and S3, respectively. The morphological analysis by optical microscopy revealed that the electrosprayed MS, in their hydrated state, have a quasi-spherical shape and compact structure. In addition, the microspheres were cross-sectioned to evaluate their internal structure. No significant porosity on the micrometer scale was observed inside the microspheres. This observation is consistent with the average droplet size (1.96 ± 0.70 μm) of the alginate/Ozoile emulsion (see Supplementary Materials), from which compact microspheres are fabricated by electrospray combined with a fast gelling in CaCl_2_ bath.

**Figure 3:**
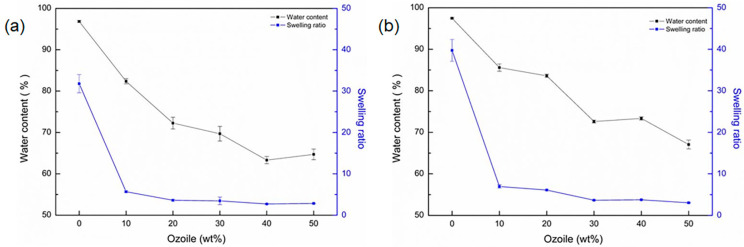
Water content and swelling ratio of (**a**) alginate microspheres containing Ozoile (0–50 wt%) without and (**b**) with chitosan coating in ultrapure water at T = 25 °C.

Figure 3 shows the swelling ratio and water content (%) of gel microspheres containing Ozoile (0–50 wt%) at room temperature (T = 25 °C). All types of microspheres have a water content above 60%. The high water content, combined with the non-toxic nature that is intrinsic with biopolymers like alginate and chitosan, suggest a good biocompatibility of the obtained microspheres [23]. In addition, in all types of microspheres (Figure 3b), chitosan coating results in increased water content due to the hydrophilic nature of the polymer. Unloaded microspheres have the highest water content and swelling when compared with those containing Ozoile, this is due to the presence of strongly hydrophilic groups such as hydroxyl (–OH) and carboxyl (–COOH). In contrast, Ozoile is characterized by a strong hydrophobic nature. Considering also that a smaller volume is available in the microspheres, as the concentration of Ozoile increases both water content and swelling ratio tend to decrease.

### 2.3. FTIR Analysis of Electrosprayed Alginate/Ozoile MS

**Figure 4:**
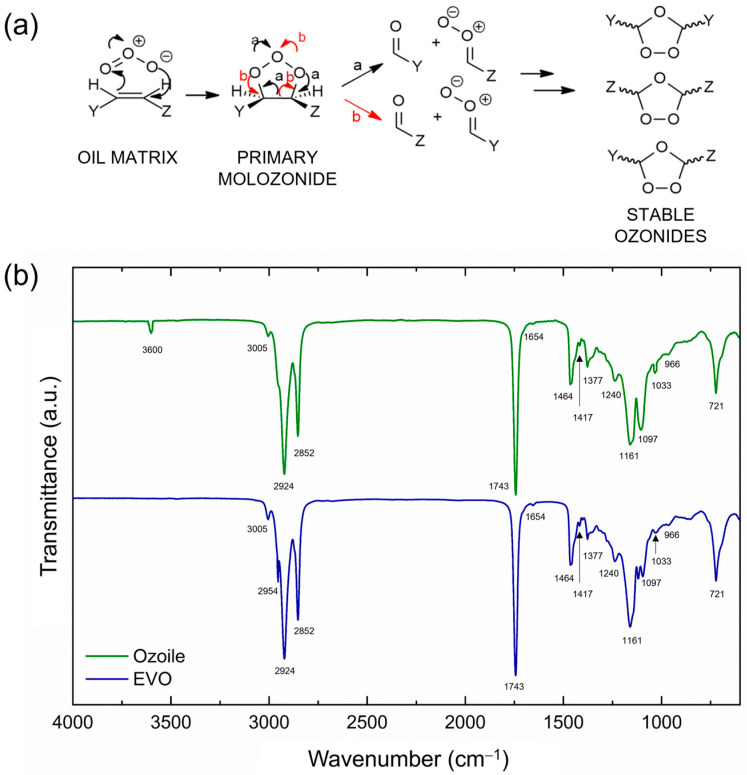
(**a**) Mechanism of formation of stable ozonides, (**b**) Comparative FTIR spectrum of EVO oil and Ozoile. Data are offset for clarity.

FTIR analysis was performed to evaluate the chemical bonds and composition of the microspheres and their successful loading with Ozoile. Additionally, a preliminary analysis was performed to compare the characteristics of EVO oil with those of Ozoile to evaluate the ozonation process (Figure 4). Figure 4a represents a schematic illustration of the process by which ozone interacts with the unsaturated triacylglycerides in EVO oil, resulting in the formation of stable ozonides. The ozonation process occurs through several steps, starting with the generation of the primary ozonide, also known as molozonide [24]. This primary ozonide breaks down into a carbonyl oxide and aldehyde compound. The subsequent combination of these two species led to the formation of more stable 1,2,4-trioxolanes, called secondary ozonides. 

The vibrational frequencies of the relevant functional groups are summarized in Table 7. In the Ozoile spectrum, a peak at 3600 cm^−1^ can be attributed to the presence of hydroxyl groups [25], while the peak at 2954 cm^−1^ exhibits reduced intensity due to ozonolysis [26] when compared to EVO oil. Additionally, the peak at 1033 cm^−1^ increases, and the peak at 3005 cm^−1^ decreases, which is associated with the reduction in unsaturated fatty acids resulting from ozone bond formation [27]. In the 1743 cm^−1^ region, no significant differences are observed between the two spectra, suggesting that the triglycerides remained unaffected by the ozonation process.

**Figure 5:**
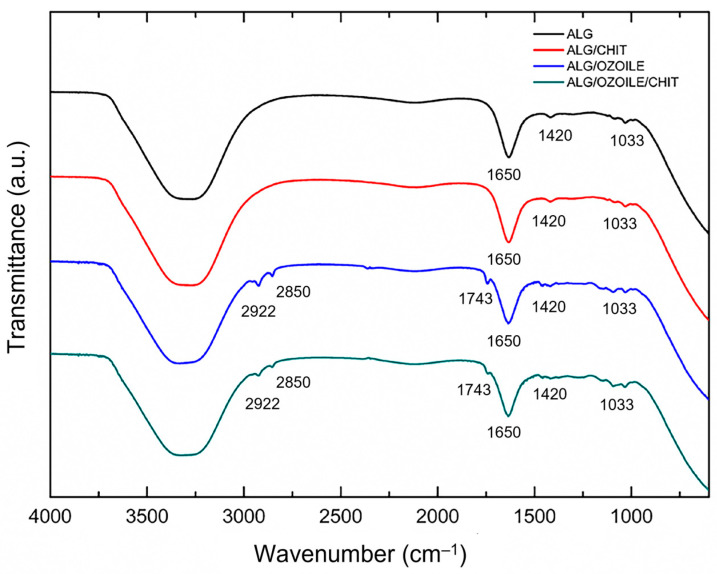
FTIR spectra of pure alginate and alginate/Ozoile microspheres prepared by electrospray (process parameters: 30 kV, 20 mL/h, 24 G needle). Data are offset for clarity.

Figure 5 shows the FTIR spectra of different types of electrosprayed gel microspheres in their hydrated state. All spectra show a broad absorption band between 3500 and 3000 cm^−1^ corresponding to the stretching of the O–H group [32]. The peaks at 1650 and 1420 cm^−1^ are related to asymmetric and symmetric stretching of the –COO^−^ group of alginate, respectively [33].

The FTIR spectra for microspheres containing Ozoile show two absorption bands around 2922 and 2850 cm^−1^, which are characteristic of the asymmetric and symmetric stretching vibrations of the methylene (–CH_2_) and methyl (–CH_3_) groups, respectively [28]. Both spectra also exhibit a peak at 1743 cm^−1^ due to the stretching of the carbonyl group (–C=O) involved in the ester bonding of triacylglycerol [31]. Finally, the peak around 1033 cm^−1^ characteristic of all four spectra can be associated with the presence of the guluronic acid units present in sodium alginate [34].

### 2.4. Thermal Analysis

**Figure 6:**
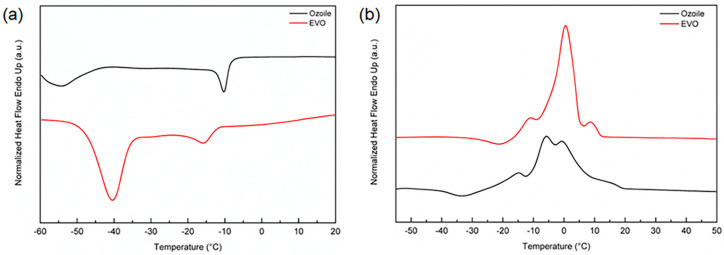
DSC thermograms during (**a**) heating and (**b**) cooling of EVO oil and Ozoile.

DSC analysis was performed to evaluate the stability and thermal behavior of EVO oil, Ozoile, and microspheres. Figure 6 shows a comparative analysis between EVO oil and Ozoile to assess the effect of oil ozonation and thermal stability. Figure 6a shows cooling from 20 °C to −60 °C at 5 °C/min to preserve the characteristic cooling profile. The thermogram revealed two distinct exothermic events [35,36]. In the EVO oil, a more significant event occurred at lower temperatures, while a less intense event appeared at higher temperatures. In contrast, in the Ozoile, the primary exothermic event occurred at higher temperatures. The exothermic event with a peak at lower temperatures has been related to the crystallization of highly unsaturated triglycerides (TAG) found in oleic acid, particularly triolein (OOO) [37]. The shape of this transition appears as a symmetric Gaussian curve, suggesting a cooperative event involving homogeneous molecules. The exothermic event at higher temperatures presents a less symmetrical shape than the event at lower temperatures, indicating the involvement of more heterogeneous molecules, identified as more saturated TAG fractions. Table 8 shows the crystallization temperatures with enthalpy changes (ΔH). Comparison of the cooling profiles reveals how the ozonation process changes the chemical composition of the oil. In particular, the peak related to highly unsaturated TAGs is more significant in EVO oil (ΔH _EVO Oil_ = −28.71; ΔH_OZOILE_ = −6.70). This result confirms that the ozonation process has occurred.

Figure 6b shows the heating thermogram from −60 °C to 50 °C at 10 °C/min. The heating profiles show multiple exothermic events in the range of −15 to 10 °C, for both EVO oil and Ozoile, attributable to the transition/rearrangement of polymorphic TAG crystals into more stable forms.

The most relevant exothermic event, T_m_ = 0.65 °C for oil and T_m_ = −5.91 °C for Ozoile, is attributable to the melting of polymorphic crystalline forms of multiple unsaturated TAG fractions (TUTAG); while the minor event at higher temperature, T_m1_ = 9.17 °C for oil and T_m1_ = −0.47 °C was attributed to the melting of more saturated TAG molecules (MSTAG and DSTAG) [37]. Both oils exhibit good teminic stability.

**Figure 7:**
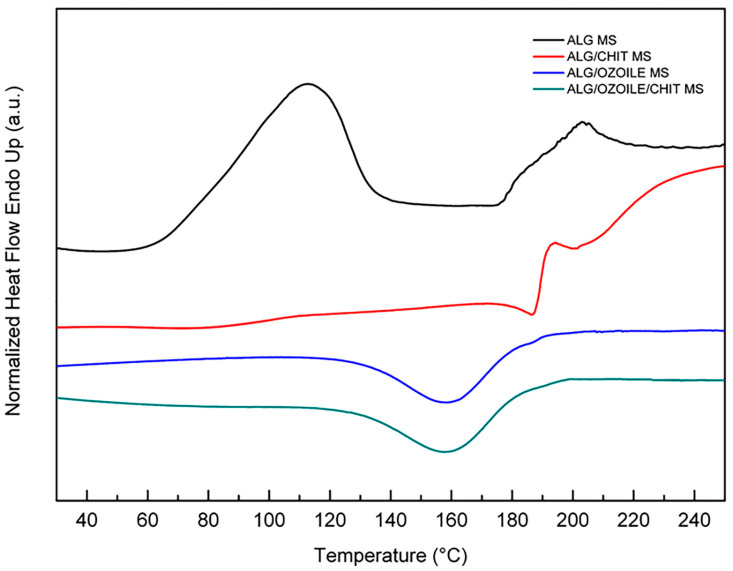
DSC thermograms of different types of microspheres.

Figure 7 shows the thermogram of microspheres with and without Ozoile in the range 25–250 °C with a heating rate of 10 °C/min. Alginate microspheres exhibit an endothermic peak, associated to water melting at a temperature of 112.8 ± 5.4 °C. After 170 °C, the degradation process starts. Chitosan-coated alginate MS does not exhibit the endothermic event, suggesting enhanced thermal stability. Ozoile-containing microspheres with and without a chitosan coating exhibit an exothermic event at a temperature of 158.6 ± 3.9 °C. This event may be related to a reaction between ozonides and alginate. In addition, microspheres containing Ozoile exhibit high thermal stability beyond the physiological range.

### 2.5. pH-Dependent Degradation of Alginate/Ozoile MS

**Figure 8:**
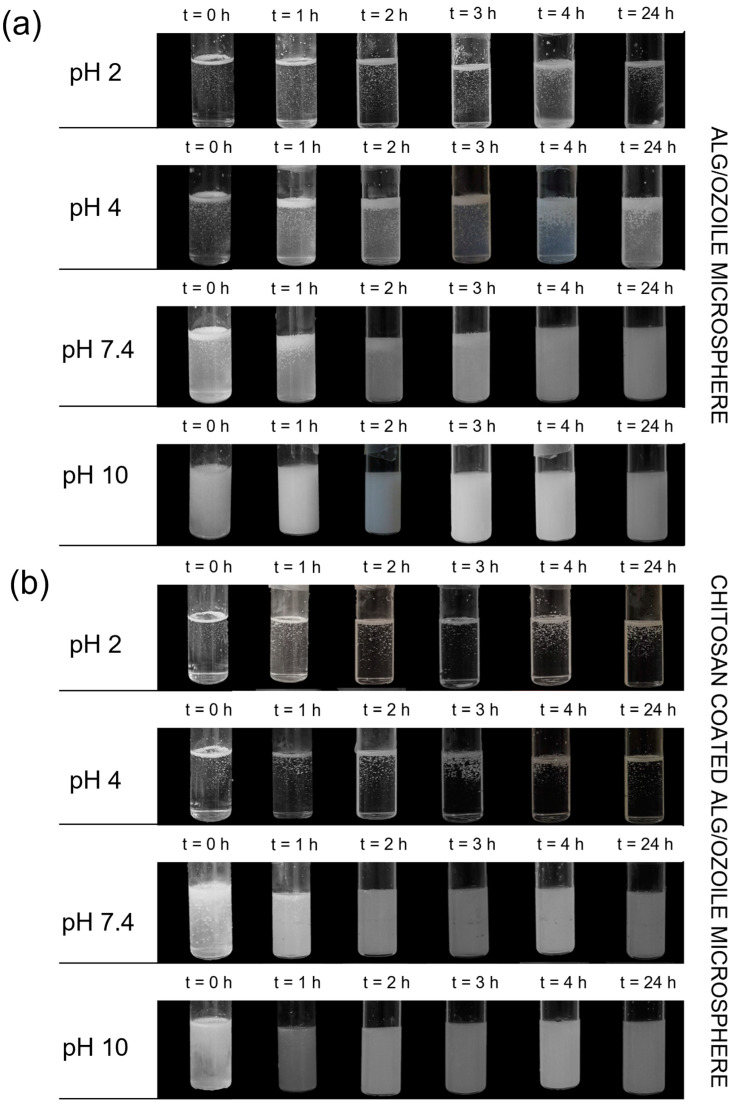
Degradation tests performed at 37 °C in PBS at different pH values (pH = 2, 4, 7.4, 10) on MS without coating (**a**) and on MS with chitosan coating (**b**). Degradation evaluated every hour for the first 4 h and after 24 h.

The degradation behavior of microspheres containing Ozoile at 20 wt% was conducted at different pH values (2, 4, 7.4, 10) in PBS at T = 37 °C. The effect of the chitosan coating on the microspheres was also evaluated, comparing the stability of MS at different pH values with and without chitosan coating (Figure 8). The degradation process is driven by the –COOH carboxyl group of the M group of alginate. In the PBS solution, the presence of Na+ ions starts an ion exchange process with the Ca^2+^ cations bound to the carboxyl groups of the alginate, leading to its dissolution. At pH = 2, the deprotonation percentage of the carboxyl group, calculated using the Henderson–Hasselbalch equation, is 2.45%, leading to a lack of electrostatic repulsion between alginate chains and thus preventing degradation. Consequently, there is poor electrostatic repulsion between the alginate chains, and degradation does not occur. Increasing the pH to 4 (where pH > pKa (Alg)), the percentage of deprotonation reaches 71.52%, enhancing electrostatic repulsion between the carboxylate anions and causing a gradual degradation of the microspheres. In contrast, chitosan-coated microspheres feature amine groups predominantly in their protonated form (NH_3_^+^) with a percentage of deprotonation of 0.35%, effectively preventing degradation. At pH = 7.4, the –COO^−^ groups significantly increase in deprotonation rate to 99.98%, and amine groups are almost entirely deprotonated at a rate of 89.91%. Consequently, degradation is delayed in chitosan-coated microspheres. However, at pH = 10, for both types of microspheres, degradation occurs almost instantaneously. Degradation tests showed the pH-responsive nature of the microspheres and the effectiveness of the chitosan coating in maintaining the stability of the microspheres in specific environments.

## 3. Conclusions

Alginate gel microspheres with pH-responsive properties were developed as carriers for lipophilic drugs, such as Ozoile. The electrospray technique followed by ionic gelation was used to fabricate the microspheres. Optimization of process parameters revealed that specific conditions, such as an applied voltage of 30 kV, a flow rate of 20 mL/h, and a 24 G needle (inner diameter 0.311 mm), were optimal for obtaining smaller and more spherical microspheres. Morphological analysis, FTIR spectroscopy, and thermal analysis provided information on the structural integrity and behavior of the microspheres. In particular, FTIR analysis confirmed the successful incorporation of Ozoile into the microspheres. Thermal analysis indicated good thermal stability of the microspheres. The degradation tests conducted at different pH values (2, 4, 7.4, 10) in PBS at 37 °C demonstrated the pH-responsive nature of the microspheres and the stabilizing effect of the chitosan coating. In summary, this research presents a promising approach for the development of targeted drug delivery systems through electrospray, particularly in applications related to the modulation of oxidative stress and the management of inflammation. The pH-responsive nature of the microspheres and the chitosan coating in regulating their degradation emphasize their potential for controlled drug release and biomedical applications. 

## 4. Materials and Methods

### 4.1. Materials

Alginic acid sodium salt, calcium chloride (CaCl_2_) dihydrate, polyethylene glycol sorbitan monooleate (Tween 80), xanthan gum, and chitosan (medium molecular weight, >75% deacetylated) were purchased from Sigma-Aldrich (Milan, Italy). Ozoile (Stable Ozonides) is a formulation obtained from a patented mixing process (EP3900821A1) in which a defined oxygen–ozone mixture is bound to olefinic bonds of fatty acids of a biological olive oil forming stable ozonides. Deionized water (resistivity 18.2 MΩ⋅cm) was produced by a Direct-Q3 UV water purification system (Millipore, Molsheim, France) and used in all preparations.

### 4.2. Fabrication of Gel Microspheres

Sodium alginate was added to ultrapure water at 1.8 wt% concentration and mixed for 2 h using a magnetic stirrer (C-MAG HS7, IKA, Staufen, Germany). Ozoile (0–50 wt%), the non-ionic surfactant Tween 80 (5 wt%), and the emulsifier xanthan gum (0.25 wt%) were added and a stable emulsion was obtained using the high-intensity ultrasound (HIU) method. The process was performed using the Handheld Ultrasonic Homogenizer (UP200Ht, Hielscher Ultrasonics GmbH, Teltow, Germany) for 20 min at 100% power. The acoustic cavitation induced by ultrasound increases the breakdown of oil droplets, facilitating the formation of stable oil/water (O/W) emulsions with small droplet sizes. The average size of droplets, for the Alg/Ozoile 20 wt% emulsion, was 1.96 ± 0.70 μm, as determined by optical analysis with ImageJ software (see Supplementary Materials, Figure S4). Gel MS were produced using the electrospray technique. The experimental setup consists of a precision syringe pump (NE-300, New York, NY, USA) filled with a polymeric emulsion, which is ejected as a spray through a needle connected to a high voltage generator ranging from 0 to 30 kV (CM-5 SIMCOION™, Lochem, The Netherlands). Specifically, the emulsion was dripped at a constant rate (20, 30 mL/h) from a collecting distance of 10 cm. Preliminary tests were conducted to establish the effect of the collecting distance (from the needle tip to the gelling bath surface) on the microsphere shape. We observed that collecting distances of less than 10 cm led to microspheres with short tails, while larger distances led to microsphere deformation due to the more energetic impact affecting droplets falling from higher heights, in agreement with the literature [38]. Two types of needles were used: 21 G, with an inner diameter of 0.514 mm, and 24 G with an inner diameter of 0.311 mm. A high voltage was applied to the needle tip and the gelling bath was fixed at 20 wt% of CaCl_2_. Optimization of electrospray process parameters, in terms of applied voltage (20, 30 kV), flow rate (20, 30 mL/h), and needle size (21 G, 24 G) was carried out to obtain microspheres with a compact shape and small size. The crosslinking duration was set at 30 min, based on reports in the literature showing that similar times were helpful to improve the microsphere sphericity [39]. Next, the MS were rinsed three times in ultrapure water and finally stored in water until further use. The chitosan-coated MS were prepared by vortex mixing in a 0.5 wt% chitosan solution for 30 min, then rinsed in deionized water. Five different mixing speeds were evaluated during the coating process from 500 to 700 rpm.

### 4.3. Morphological and Swelling Analyses

The morphology, shape, and particle size of microspheres were assessed using a DinoLite—AM7915MZT digital microscope coupled with the powerful DinoCapture 2.0 software. Further analysis was conducted using ImageJ software (version 1.53). ImageJ provided a robust platform for the automatic evaluation of microsphere diameter, enhancing the efficiency and accuracy of the analysis. To analyze the microspheres, the microscope images were first scanned to grayscale by bandpass filter mode (8-bit). In the following step, the monochrome images were put on a threshold, and finally, to analyze the microspheres the “Analyze particles” tool was used. A population of 50 samples was analyzed for each type of microsphere. To quantify the degree of sphericity, the sphericity factor (SF) was determined using the following equation [40]:

(1) $$\mathrm{SF}\;=\frac{D_{1}-D_{2}}{D_{1}+D_{2}}$$

where D_1_ represents the larger diameter of the beads, while D_2_ is the smaller one. When SF ≤ 0.05, the shape of the matrix can be approximated to a sphere. Statistical analyses were performed using GraphPad Prism v.10.0.2 software. Data were analyzed using the analysis of three-way variance (ANOVA) test to determine the effects of electrospray process parameters on the mean diameter size and sphericity of the microspheres. The significance was determined at the 95% confidence level.

To evaluate the swelling ratio and water content, the gel microspheres were first completely hydrated in deionized water and then dried in an oven at 50 °C for 18 h. The swelling ratio and water content were calculated using the following equations [41]:

(2) $$\mathrm{Swelling}\;\mathrm{Ratio}\;\left(\%\right)=\frac{W_{\mathrm{swollen}}}{W_{\mathrm{dry}}}\times 100$$

(3) $$\mathrm{Water}\;\mathrm{content}\;(\%)=\frac{W_{\mathrm{swollen}}-W_{\mathrm{dry}}}{W_{\mathrm{swollen}}}\times 100$$

W_swollen_ is the weight of the gel in the equilibrium swelling state, and W_dry_ is the weight of the fully dried gel. The test was repeated on 5 samples for microspheres containing Ozoile (0–50 wt%), with and without chitosan coating, and the results were averaged.

### 4.4. FTIR Analysis

Fourier transform infrared (FTIR) spectra of the EVO oil, Ozoile, and microspheres were recorded using a Nicolet^TM^ Summit/Everest^TM^ Spectrometer (Thermo Fisher Scientific, Waltham, MA, USA) equipped with a zinc selenide attenuated total reflectance (ATR) accessory. IR spectra were acquired in the region of 4000–600 cm^−1^, with a resolution of 4 cm^−1^, and each spectrum was acquired by averaging 64 scans. To account for the background interference from the surrounding air, the data were corrected by subtracting an air background spectrum. In the FTIR spectra of the oils, the peaks were evaluated according to their intensity as weak, moderate, or strong [42].

### 4.5. Thermal Characterization

The stability and thermal properties of EVO oil, Ozoile, and loaded and unloaded microspheres were investigated by differential scanning calorimetry (DSC). A Pyris DSC 8500 instrument (PerkinElmer, Waltham, MA, USA) was used. DSC samples were sealed in aluminum pans with lids and measurements were performed under a constant flow of nitrogen (40 mL/min). An identical empty cell was taken as a reference. For the determination of the thermal behavior of the oils, the samples (3 samples, ~5 mg) were tested by cooling from 25 °C to −60 °C at 5 °C/min and heating from −60 °C to 50 °C at 10 °C/min. The stability of the swollen microspheres (3 samples, ~25 mg) was evaluated from 25 °C to 250 °C with a heating rate of 10 °C/min.

### 4.6. Degradation Test

Degradation tests were conducted on microspheres containing Ozoile at 20 wt% with and without chitosan coating to evaluate the pH response and stabilizing effect of the coating. The tests were performed on 60 mg of MS swollen and immersed in 10 mL of phosphate-buffered saline (PBS) solution. The microspheres were tested at different pH levels (2, 4, 7.4, 10) at a temperature of 37 °C, and their behavior was monitored hourly in the first 4 h and after 24 h. To understand the phenomenon of degradation as pH changes, the deprotonation rate of carboxyl groups was calculated using the Henderson–Hasselbalch equation [43]:

(4) $$\%\;\mathrm{deprotonation}=\frac{10^{\left(\mathrm{pH}-{\mathrm{pK}}_{a}\right)}}{10^{\left(\mathrm{pH}-{\mathrm{pK}}_{a}\right)}+1}\times 100$$

where pK_a_ is the acid dissociation constant of the carboxylic group involved. This formula expresses the relationship between the pH of the environment and the percentage of deprotonated carboxylic groups at that specific pH.

## Figures and Tables

**Table 1:** Mean diameter of alginate/Ozoile microspheres (Ozoile 20 wt%) fabricated by electrospray process with different parameters. Needle size refers to the inner diameter.

Flow Rate (mL/h)	Sample	Needle Size 0.311 mm (24 G)		Needle Size 0.514 mm (21 G)	
		25 kV	30 kV	25 kV	30 kV
		Mean Diameter (μm)	Mean Diameter (μm)	Mean Diameter (μm)	Mean Diameter (μm)
20	No coating	311 ± 25	279 ± 21	456 ± 21	375 ± 37
	Coating	265 ± 24	249 ± 27	359 ± 45	331 ± 37
30	No coating	353 ± 32	328 ± 39	705 ± 50	428 ± 18
	Coating	306 ± 19	268 ± 28	663 ± 61	372 ± 24

**Table 2:** ANOVA results: evaluation of the effects of the electrospray process parameters on microsphere size.

Source of Variation	% of Total Variation	SS	DF	MS	F Ratio	*p* Value	Significant?
Needle size	44.38	3,001,037	1	3,001,037	F = 2965	<0.0001	Yes
Flow rate	14.22	961,969	1	961,969	F = 950.3	<0.0001	Yes
Applied voltage	15.96	1,079,521	1	1,079,521	F = 1066	<0.0001	Yes
Needle size × Flow rate	4.082	276,045	1	276,045	F = 272.7	<0.0001	Yes
Needle size × Applied voltage	8.436	570,478	1	570,478	F = 563.6	<0.0001	Yes
Flow rate × Applied voltage	3.235	218,790	1	218,790	F = 216.1	<0.0001	Yes
Needle size × Flow rate × Applied voltage	3.817	258,115	1	258,115	F = 255.0	<0.0001	Yes

SS = Sum of squares; DF = Degrees of freedom; MS = Mean squares.

**Table 3:** Sphericity factor (SF) of alginate microspheres containing Ozoile (20 wt%), with and without chitosan coating, obtained by electrospray with different process parameters.

Flow Rate (mL/h)	Sample	Needle Size 0.311 mm (24 G)		Needle Size 0.514 mm (21 G)	
		25 kV	30 kV	25 kV	30 kV
		SF	SF	SF	SF
20	No coating	0.041 ± 0.035	0.025 ± 0.021	0.019 ± 0.015	0.040 ± 0.029
	Coating	0.044 ± 0.033	0.038 ± 0.026	0.056 ± 0.044	0.039 ± 0.034
30	No coating	0.023 ± 0.018	0.019 ± 0.014	0.012 ± 0.010	0.029 ± 0.021
	Coating	0.036 ± 0.051	0.040 ± 0.038	0.048 ± 0.035	0.040 ± 0.031

**Table 4:** ANOVA results: evaluation of the effects of the electrospray process parameters on the sphericity of microspheres.

Source of Variation	% of Total Variation	SS	DF	MS	F Ratio	*p* Value	Significant?
Needle size	0.1797	0.0004000	1	0.0004000	F = 0.8437	0.3589	No
Flow rate	4.954	0.01103	1	0.01103	F = 23.25	<0.0001	Yes
Applied voltage	0.9099	0.002025	1	0.002025	F = 4.271	0.0394	Yes
Needle size × Flow rate	0.1011	0.0002250	1	0.0002250	F = 0.4746	0.4913	No
Needle size × Applied voltage	9.447	0.02103	1	0.02103	F = 44.34	<0.0001	Yes
Flow rate × Applied voltage	0.1797	0.0004000	1	0.0004000	F = 0.8437	0.3589	No
Needle size × Flow rate × Applied voltage	0.7189	0.001600	1	0.001600	F = 3.375	0.0670	No

SS = Sum of squares; DF = Degrees of freedom; MS = Mean squares.

**Table 5:** Effect of stirring speed, during application of chitosan coating, on size and swelling properties of Alg/Ozoile 20 wt% microspheres.

rpm	Mean Diameter (μm)	Water Content (%)	Swelling Ratio
500	305 ± 28	84.18 ± 0.48	6.33 ± 0.19
550	295 ± 24	79.75 ± 0.39	4.94 ± 0.09
600	290 ± 19	81.85 ± 0.51	5.51 ± 0.15
650	262 ± 30	80.52 ± 0.71	5.14 ± 0.19
700	249 ± 27	82.19 ± 0.92	5.62 ± 0.31

**Table 6:** Mean diameter of microspheres containing Ozoile (0–50 wt%) with and without chitosan coating.

Ozoile (wt%)	Mean Diameter (μm)	
	No Coating	Coating
0	296 ± 27	277 ± 18
10	257 ± 15	238 ± 21
20	279 ± 21	249 ± 27
30	286 ± 12	267 ± 22
40	299 ± 18	284 ± 30
50	326 ± 32	313 ± 28

**Table 7:** Assignment of main peaks in the FTIR spectra of EVO oil and Ozoile.

Frequency (cm^−1^)	Functional Group Vibration	Peak Intensity		Ref.
		EVO Oil	Ozoile	
3600	O–H stretching	-	m	[25]
3005	=C–H stretching	m	w	[27]
2954	Asymmetric stretching vibration of methyl (–CH_3_) group	m	w	[26]
2924 and 2852	Asymmetric and symmetric stretching vibration of methylene (–CH_2_) group	m	s	[28]
1743	Carbonyl (C=O) from the ester linkage of triacylglycerol	s	s	[29]
1654	cis C=C	s	m	[27]
1464	Bending vibrations of the CH_2_ and CH_3_ aliphatic groups	s	s	[27]
1417	Rocking vibrations of CH bonds of cis-disubstituted alkenes	m	w	[30]
1377	Symmetric bending vibrations of CH_3_ groups	w	s	[30]
1240	vibrations of stretching mode from the C–O group in esters	w	m	[30]
1161	vibrations of stretching mode from the C–O group in esters	w	s	[30]
1097	–CH bending and –CH deformation vibrations of fatty acids	w	w	[31]
1033	C–O stretching	w	m	[27]
966	bending vibration of CH functional groups of isolated trans-olefin	w	w	[30]
907	Bending vibration of cis –HC=CH–	w	-	[31]
721	Overlapping of the methylene (–CH_2_) rocking vibration and to the out of plane vibration of cis-disubstituted olefins	s	s	[30]

w = weak; m = moderate; s = strong.

**Table 8:** Peak temperature (T_C_) and enthalpy (ΔH) values of EVO oil and Ozoile obtained from DSC analysis.

Sample	T_C_	ΔH	T_C_^′^	ΔH
EVO Oil	−40.35 ± 0.25	−28.71 ± 1.06	−21.22 ± 0.70	−2.69 ± 0.85
Ozoile	−54.16 ± 0.18	−6.70 ± 0.36	−11.72 ± 1.60	−4.83 ± 0.97

## Data Availability

All data and materials are available on request from the corresponding author. The data are not publicly available due to ongoing researches using a part of the data.

## Supplementary Materials

The following supporting information can be downloaded at: https://www.mdpi.com/article/10.3390/gels10010052/s1, Figure S1: Bar graph showing three-way ANOVA analysis for the microsphere mean diameter considering the electrospray process parameters (applied voltage, flow rate, needle size); Figure S2: Histograms of the diameter distribution of alginate microspheres with different concentrations of Ozoile prepared by electrospray process (30 kV, 20 mL/h, 24 G needle); Figure S3: Histograms of the diameter distribution of chitosan-coated alginate microspheres with different concentrations of Ozoile prepared by electrospray process (30 kV, 20 mL/h, 24 G needle); Figure S4: (a,b) Light microscope images of Alg/Ozoile emulsion at 20 wt% of Ozoile with different magnifications; (c) binarization of (b) using ImageJ software.
