# Supplementary material for: Fabrication of Alginate/Ozoile Gel Microspheres by Electrospray Process
Source: Gels. 2024 Jan 11;10(1):52. doi: 10.3390/gels10010052 (PMC10815707; doi:10.3390/gels10010052)
Supplement: Supplementary file 1 [file gels-10-00052-s001.zip › gels-2764911-supplementary.pdf]

Supplementary Materials

Fabrication of Alginate/Ozoile Gel Microspheres by Electrospray Process

Gianluca Ciarleglio [1,2], Tiziana Russo [1,†], Elisa Toto [1] and Maria Gabriella Santonicola [1,*]

1 Department of Chemical Engineering Materials Environment, Sapienza University of Rome, Via del Castro Laurenziano 7, 00161 Rome, Italy; gianluca.ciarleglio@uniroma1.it (G.C.); trusso@ibecbarcelona.eu (T.R.); elisa.toto@uniroma1.it (E.T.)
2 Erbagil s.r.l., Via Luigi Settembrini 13, 82037 Telese Terme, Italy
* Correspondence: mariagabriella.santonicola@uniroma1.it
† Present address: Institute for Bioengineering of Catalonia, C/Baldiri Reixac 10-12, 08028 Barcellona, Spain.

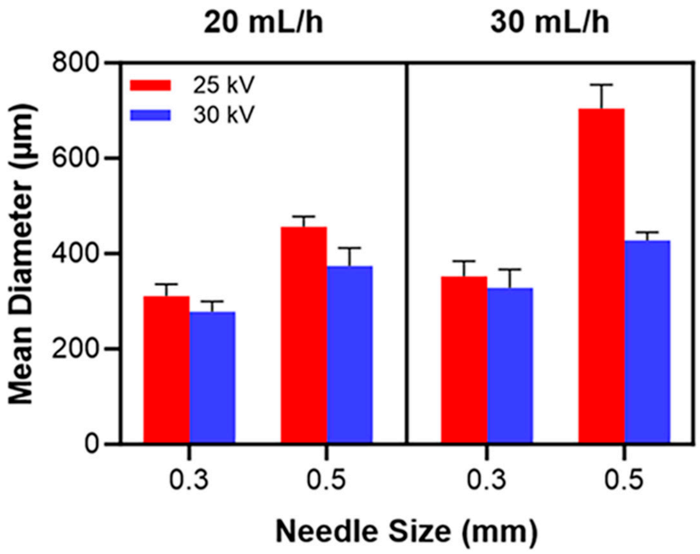

Figure S1. Bar graph showing three-way ANOVA analysis for the microsphere mean diameter considering the electrospray process parameters (applied voltage, flow rate, needle size).

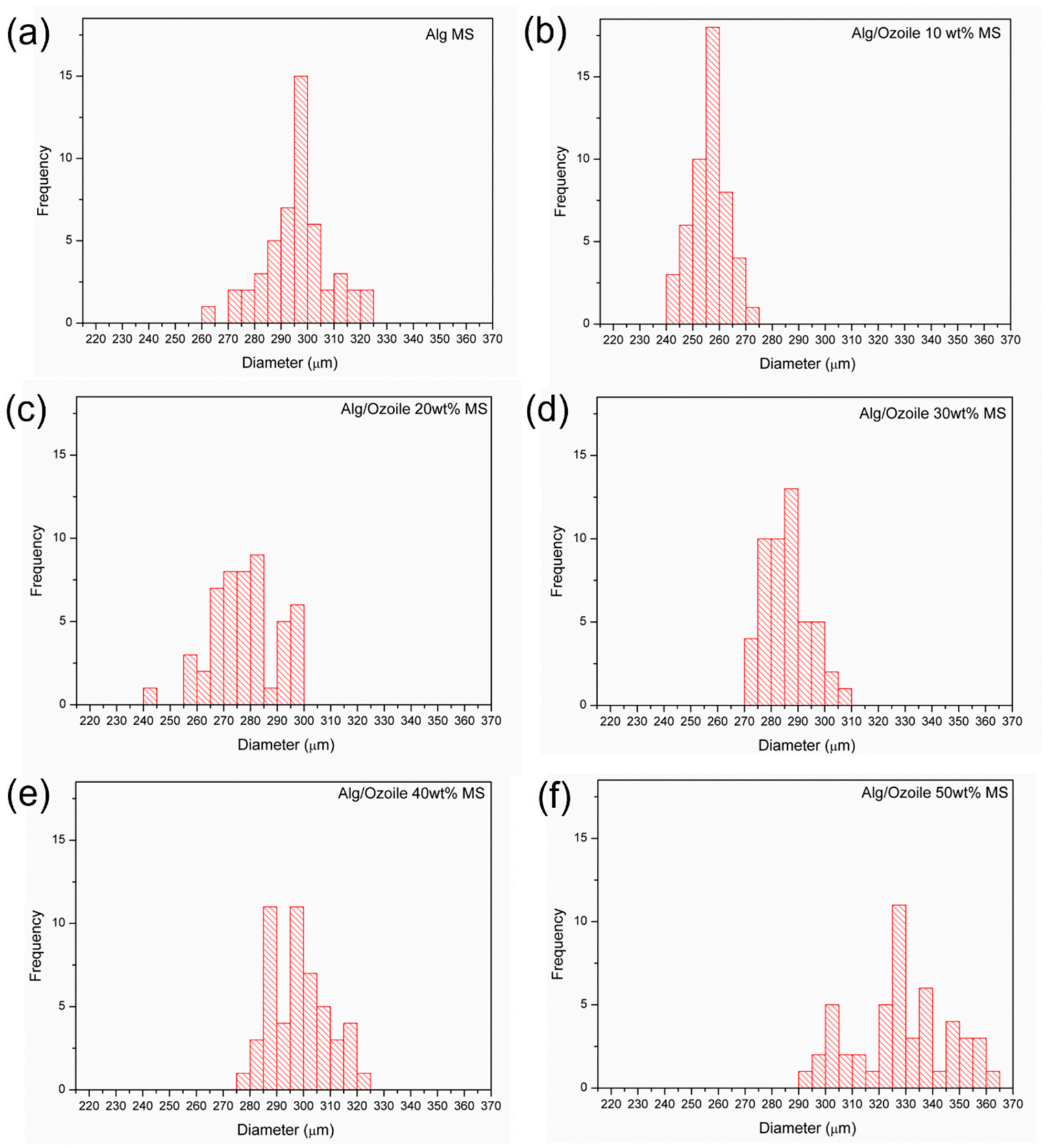

Figure S2. Histograms of the diameter distribution of alginate microspheres with different concentrations of Ozoile prepared by electrospray process (30 kV, 20 mL/h, 24 G needle).

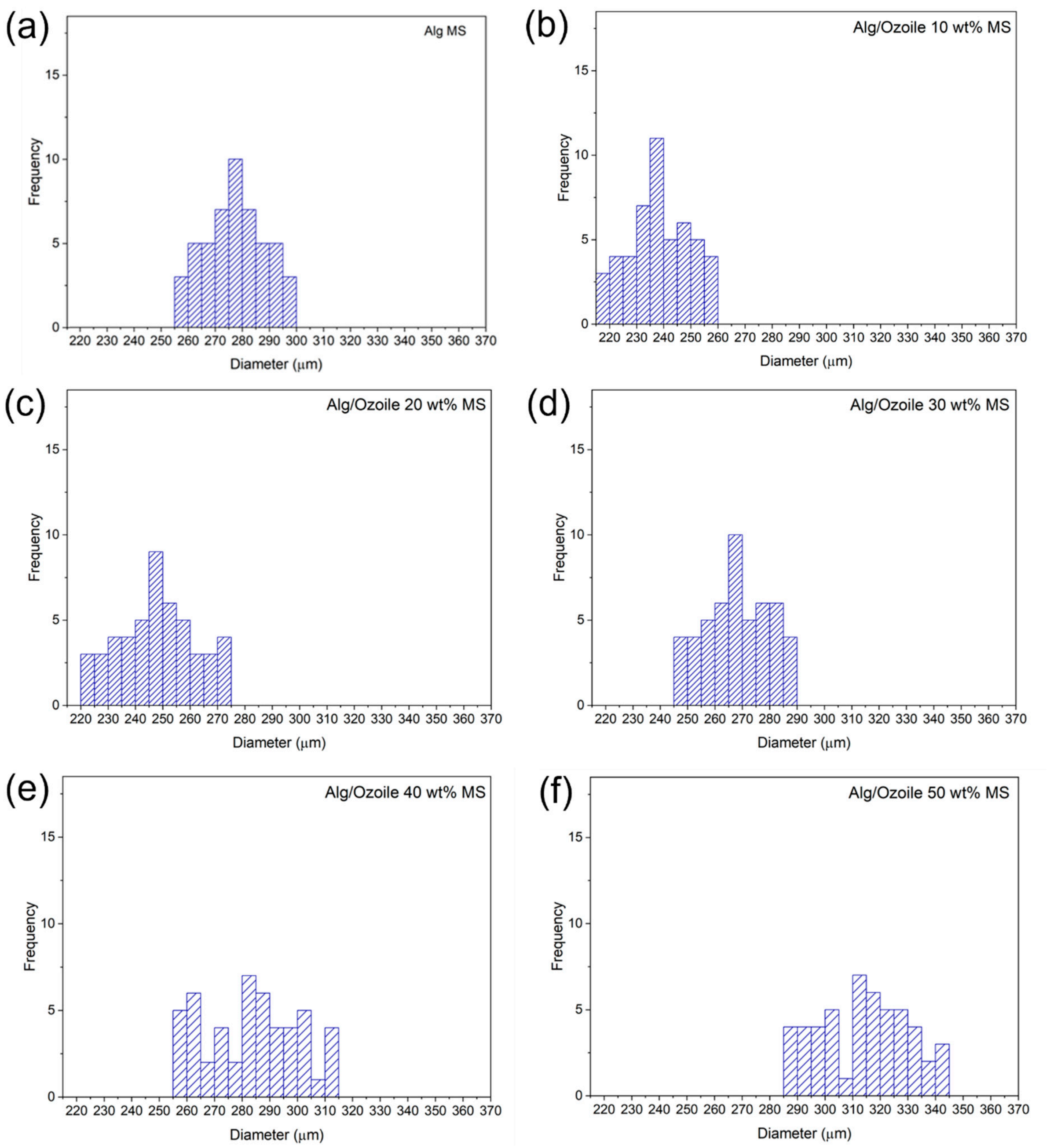

Figure S3. Histograms of the diameter distribution of chitosan-coated alginate microspheres with different concentrations of Ozoile prepared by electrospray process (30 kV, 20 mL/h, 24 G needle).

Determination of emulsion size by light microscopy

The size analysis of the Alg/Ozoile emulsion prepared by high-intensity ultrasound method was performed using the Leica DMLP microscope, specifically on emulsions containing 20 wt% of Ozoile (Figure S4). The emulsion was analyzed immediately after sonication, at room temperature. A drop of distilled water and then a drop of emulsion were dispensed on a concave microscope slide, followed by careful spreading and covering with a glass coverslip. The acquired images were analyzed using ImageJ software and the "Analyze Particles" tool. Approximately 3500 droplets were analyzed. The image analysis results revealed an average diameter of the Ozoile droplets in the emulsion of 1.96 ± 0.70 µm.

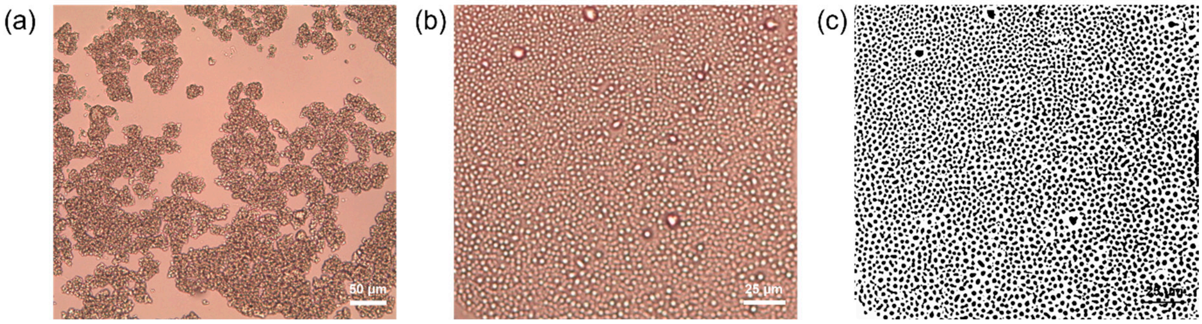

Figure S4. (**a**,**b**) Light microscope images of Alg/Ozoile emulsion at 20 wt% of Ozoile with different magnifications; (**c**) binarization of (b) using ImageJ software.
